# Low-Cost Ceramic Membrane with Concentrated Pore Size Distribution for Flue Gas Moisture Recovery

**DOI:** 10.3390/membranes16100333

**Published:** 2026-10-04

**Authors:** Tao Li, Zheng Zhang, Tengfei Li, Jiguang Huang, Heng Zhang, Haiping Chen

**Affiliations:** 1 School of Energy, Power and Mechanical Engineering, North China Electric Power University, Beijing 102206, China; 2 Jionto Energy Investment Co. Ltd., Shijiazhuang 050051, China; 3 Beijing Key Laboratory of Pollutant Monitoring and Control in Thermoelectric Production Process, North China Electric Power University, Beijing 102206, China

**Keywords:** ceramic membrane, low cost, pore size distribution, flue gas, moisture recovery

## Abstract

Transport membrane condenser with ceramic membrane has been proven to be effective in recovering water from industrial flue gas. A concentrated pore size distribution of the ceramic membrane assures high recovery performance and water quality. In this study, a low-cost ceramic membrane with concentrated pore size distribution is prepared using a coal fly ash as the main material. The effects of particle content and particle size on membrane are studied. Flue gas moisture recovery performance of the membrane is also investigated. The membrane prepared by small particle exhibits smaller pore size and a narrower pore size distribution. The smallest average pore size is 0.11 μm and approximately 90% of the measured pores fall within the most probable pore-size interval. The moisture recovery flux is 1.81 kg·m^−2^·h^−1^ while the moisture recovery rate is 70.5%. This study indicates the feasibility of the low-cost ceramic membrane with concentrated pore size distribution for the application of flue gas moisture recovery.

## 1. Introduction

The ceramic membrane, with its excellent chemical stability, thermal stability, high mechanical strength and long service life [1], has extensive applications in fields such as gas separation, food industry, biopharmaceuticals, oil–water separation and wastewater treatment [2,3]. Ceramic membranes are increasingly applied at industrial scale, and comparative techno-economic assessments have shown that their longer service life and their higher permeability can offset their higher initial cost [4,5]. In recent years, transport membrane condensers (TMC), with ceramic membranes as their core components, have been demonstrated to effectively capture and recover moisture from flue gas [6]. Compared with other traditional technologies for recovering moisture from flue gas, such as condensation methods [7,8], solution absorption methods [9,10], and spray methods [11,12,13], the ceramic membrane method can adapt to higher temperatures and acidic conditions for a longer time, and avoid direct contact between the cooling medium and the flue gas, achieving effective separation of pollutants in the flue gas from the condensed water, and obtaining water with high quality.

Up to now, however, the industrial-scale recovery of moisture from flue gas is still based mainly on these traditional methods, in which the flue gas is in direct contact with the cooling or absorbing medium, so that the acid gases and the particulate matter carried by the flue gas are transferred into the recovered water and the condensate generally needs further treatment before it can be reused [9,11]. The TMC avoids this contact, but it has so far been demonstrated mainly at the pilot scale [14,15]. The TMC has great potential in solving the water shortage problem in regions with heavy industries such as metallurgy and power [14]. Freshwater scarcity affects about two-thirds of the global population for at least one month of the year [16], so recovering water on site from industrial flue gas is of practical interest. The concept of the TMC has also been extended to other module designs: a flat-sheet ceramic membrane condenser has been used to recover heat and water from a saturated gas stream, and a water recovery rate of 84.1% was obtained under optimized operating conditions [17]. These results indicate that the recovery performance of a membrane condenser is governed mainly by the pore structure of the membrane and by the operating conditions rather than by the geometry of the module. Currently, the research on TMC mainly focuses on optimizing the structural parameters of the membrane components [18], regulating the operating conditions of the system [19], and constructing thermal and mass transfer models [20]. These efforts lay an important foundation for system design optimization and economic operation improvement [21].

The membrane pore size is a key structural parameter affecting the moisture recovery performance of the TMC. Ceramic membranes with smaller pore sizes show unique advantages in flue gas moisture recovery. On one hand, based on the Kelvin equation, the capillary condensation effect may occur when pore size of ceramic membrane lies in the range of 2–50 nm, which significantly enhances the water vapor condensation rate and heat transfer efficiency [22]. Li et al. demonstrated in a pilot study with 10,000 m^3^·h^−1^ flue gas that the maximum moisture recovery flux of membranes with 0.4 nm pore size reached 26.6 kg·m^−2^·h^−1^, which was 27.9% higher than that of 1 μm pore size [15]. On the other hand, reducing the membrane pore size helps improve the water quality. Cheng et al. found that the smaller the membrane pore size, the lower the conductivity of the cooling water at the outlet of the component, indicating that reducing the pore size can effectively block the transmembrane permeation of soluble pollutants in the flue gas [23]. However, too small a pore size will cause a thick liquid film of condensate to form on the flue gas side, significantly increasing the mass transfer resistance and thermal resistance, thus reducing the recovery flux and increasing energy consumption [24]. Therefore, narrowing the pore size distribution of the ceramic membrane, while ensuring a high water recovery flux, can improve the water quality and is an important direction for optimizing the performance of the TMC.

Ceramic membranes with a narrow pore size distribution have therefore been pursued intensively in recent years. Gu et al. obtained asymmetric SiC membranes with a narrow pore size distribution on reaction-bonded supports by matching the particle size of the coating powder to the pore structure of the support [25], and Yan et al. prepared an alumina-based membrane with an average pore size of 83 nm in which 98.3% of the pore population lies in a single interval [26]. These membranes, however, were developed for the separation of oil-in-water emulsions, and the pore size distribution of a ceramic membrane intended for flue gas moisture recovery, where the membrane operates at an elevated temperature and in the presence of acid gases, has rarely been investigated. Defect-free separation layers of this kind are almost always produced by dip-coating, and several strategies have been used to prevent the coating suspension from penetrating the pores of the support and to suppress the formation of pinholes: the dip-coating step can be modified so that pinhole-free layers are obtained [27], the layer can be deposited in a single coating operation [28,29], it can be cured with ultraviolet light before drying [30], or the separation layer can be co-sintered with the support in a single thermal treatment, which removes one firing step and reduces the energy consumption of fabrication [31].

Reducing the manufacturing cost of ceramic membranes is another key approach to promoting the industrial application of TMC. Li et al. showed that the purchase cost of ceramic membrane materials can account for more than 50% of the initial cost of the entire moisture recovery system [14]. Using industrial waste coal fly ash (CFA) to replace high-purity metal oxides (such as alumina, zirconia) to prepare ceramic membranes is considered a feasible strategy to reduce the manufacturing cost [32]. CFA, as the main solid waste from coal-fired power plants, is widely available and inexpensive, and its chemical composition (mainly SiO_2_ and Al_2_O_3_) is similar to that of the raw materials of ceramic membranes [33]. It is also one of the largest industrial solid wastes generated in China, and processes for recovering alumina from it have been developed to the industrial scale [34], which further supports its use as a membrane precursor. Existing studies have confirmed that CFA-gypsum-based ceramic membranes can achieve a porosity of 42.7% and a flexural strength of 43.6 MPa, with a performance comparable to commercial membrane materials [35]. Low-cost ceramic membranes have also been fabricated from coal fly ash blended with natural sand, for which a pore size of 0.64 μm and a porosity of 34.7% were obtained at a sintering temperature of only 1000 °C [36]. These studies confirm that CFA is technically suitable as the main raw material of a ceramic membrane; nevertheless, they are aimed at the treatment of liquid streams, and none of them has yet produced a CFA-based membrane whose pore size distribution is concentrated enough for the recovery of moisture from flue gas. CFA-based mineral supports have also been characterized for microfiltration and ultrafiltration [37], and CFA has been combined with alumina to prepare membrane supports [38] and composite membranes [39], with kaolin [40], and with dolomite to form anorthite-cordierite supports [41]. All of these membranes, however, were developed for the treatment of liquid streams.

Although low-cost CFA-based ceramic membranes have significant economic advantages, the particle size distribution of CFA without long-term grinding and multiple sieving is wide, and the average pore size of the support material prepared from it is large and the pore size distribution is broad, making it difficult for the recovered water to meet the industrial reuse standards [42]. Using the solid-state sintering method to prepare ceramic membranes to reduce pore size and narrow the pore size distribution is an effective way to solve this problem. Among the various parameters involved in the membrane preparation, the sintering temperature, the shape and size of the separation-layer-forming particles all affect the pore size of the membrane. Among these factors, the influence of particle size is the most significant [43,44]. However, at present, studies on systematically exploring the influence of different particle contents and sizes on the pore size of ceramic membranes based on CFA-based support are still lacking [45,46]. At the same time, most existing research on CFA-based membranes focuses on the influence of preparation parameters on membrane properties such as pore size, porosity, and mechanical strength, lacking application research for flue gas moisture recovery. The moisture recovery performance of CFA-based membranes with concentrated pore size needs to be experimentally investigated.

Based on the research gap in the CFA-based membranes with concentrated pore size in the application of flue gas moisture recovery, this work prepared a ceramic support using low-cost CFA as the raw material, and added a separation layer on it to prepare ceramic membranes to explore the influence of different particle contents and sizes on the pore size of the ceramic membranes. At the same time, the flue gas moisture recovery performance of the CFA-based membranes was experimentally investigated, proving the feasibility of the low-cost ceramic membranes with concentrated pore size in the application of flue gas moisture recovery, with the aim of significantly reducing the investment cost of the ceramic membrane flue gas moisture recovery system.

## 2. Experiment

### 2.1. Materials

Coal fly ash, provided by Datang Tocto Power Plant of China (Hohhot, China), was used as the main raw material for the CFA-based ceramic supports. The CFA was pretreated with mesh sieves to obtain an average particle size of 1.28 μm. The chemical composition of the sieved powder determined by X-ray fluorescence is SiO_2_ (48.6 wt.%), Al_2_O_3_ (33.5 wt.%), Fe_2_O_3_ (4.2 wt.%), CaO (3.6 wt.%), and TiO_2_ (1.5 wt.%). Carboxymethyl cellulose (CMC, CAS 9004-32-4, assay ≥ 99.0%) and dextrin (CAS 9004-53-9, assay ≥ 99.0%), as the organic binder and pore-forming agent, were both purchased from Tianjin Huasheng Chemical Reagent Co., Ltd. (Tianjin, China). Glycerin (CAS 56-81-5, assay ≥ 99.5%), obtained from Shanghai Aladdin Biochemical Technology Co., Ltd. (Shanghai, China), was used to reduce the sintering temperature and improve plasticity of the ceramic paste. The separation-layer-forming α-Al_2_O_3_ particles were supplied by Zhejiang Lixie Instrument Equipment Co., Ltd. (Yiwu, China). Sodium hexametaphosphate (SHMP, CAS 10124-56-8, assay ≥ 99.0%) and polyvinyl alcohol (PVA, CAS 9002-89-5, degree of hydrolysis 99%, weight-average molecular weight 75,000), functioning as the dispersant and the binder of the separation-layer-forming suspension respectively, were both purchased from Shanghai Aladdin Biochemical Technology Co., Ltd. (Shanghai, China).

### 2.2. Preparation of CFA-Based Supports and Membranes

The extrusion method is applied to prepare tubular CFA-based supports. The preparation steps are as follows: A certain proportion of CFA, dextrin, and CMC is mixed and grinded in a ball mill for 30 min. Then glycerol and deionized water are added and stirred in a mixer for 30 min. The slurry is thoroughly stirred before undergoing vacuum molding 3–4 times to remove the air inside the slurry. After 24 h of aging, the slurry is extruded by an extrusion machine to form a tubular green body. The green body is dried for 24 h and then sintered at 1150 °C to obtain the final ceramic support. The detailed preparation steps of the support are reported in our previous work [47].

**Figure 1:**
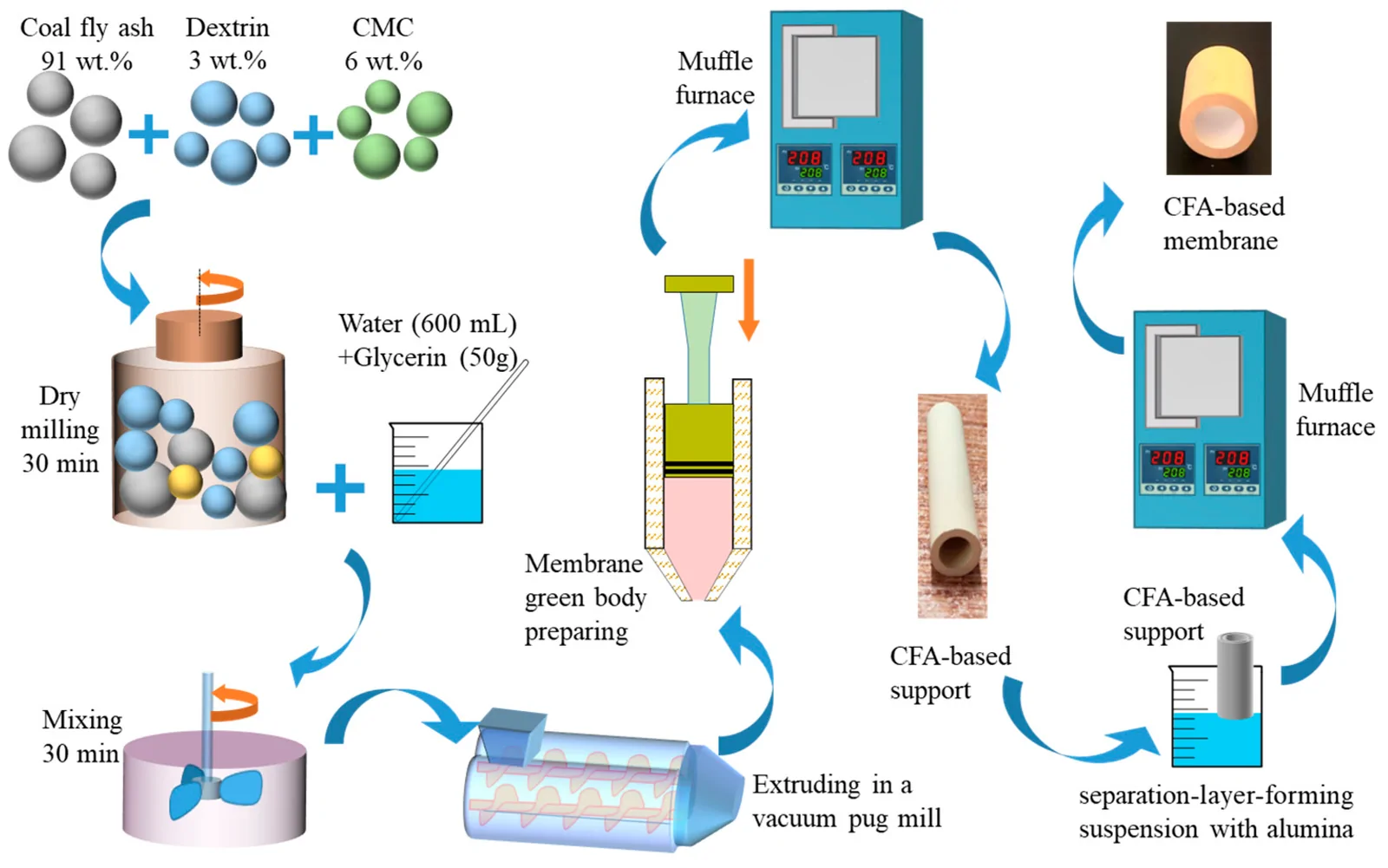
Schematic diagram of CFA-based membrane.

Based on the preparation of the support, five different particle sizes (with average particle sizes of 0.58, 0.82, 1.11, 1.85, and 2.86 μm) of alumina powder were used to coat and form a separation layer, to elaborate the ceramic membrane. The preparation process for the separation layer includes preparing a separation-layer-forming suspension containing five concentrations of alumina (5 wt.%, 10 wt.%, 15 wt.%, 20 wt.%, 25 wt.%), 0.5 wt.% sodium hexametaphosphate, and 0.5 wt.% polyvinyl alcohol. The suspension is ultrasonically treated for 20 min and stirring for 30 min. A liquid film is built on the support using the dip-coating method. The support is kept rotating at a constant speed of 10 r·min^−1^ and dried at room temperature for 30 h. The support is finally sintered at 1100 °C for 3 h at a constant temperature to obtain the ceramic membrane. Figure 1 is the schematic diagram of the CFA-based membrane. The geometrical parameters of CFA-based supports and membranes are shown in Table 1.

### 2.3. Characterization

**Figure 2:**
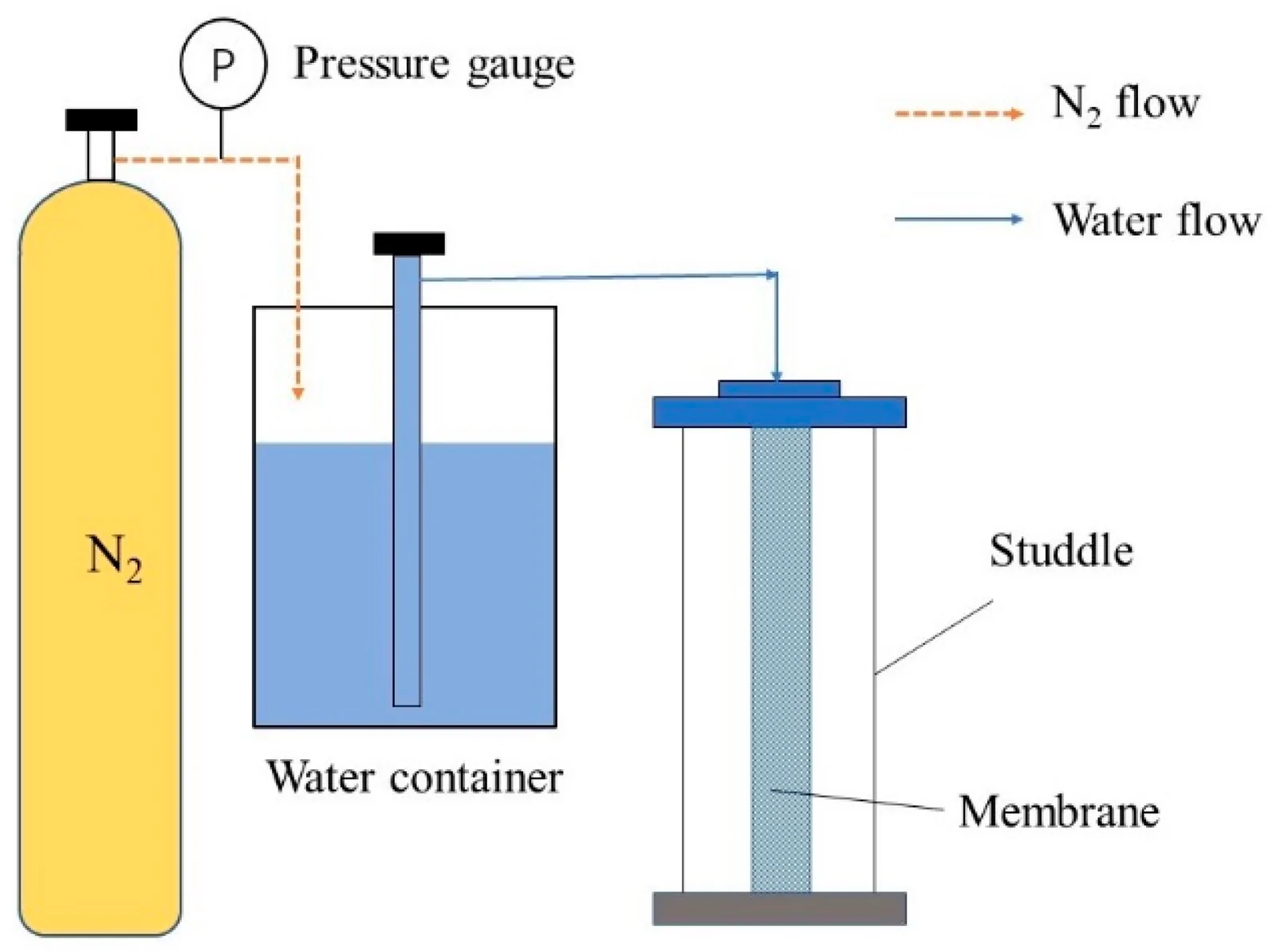
Schematic diagram of test platform for water permeate flux.

A scanning electron microscope (Quanta 400 FEG, FEI, Hillsboro, OR, USA) is used to observe the morphology of alumina powder, CFA-based supports, and membranes. The particle size distribution of alumina powder was statistically analyzed by software ImageJ (Version 1.54t, National Institutes of Health, Bethesda, MD, USA). The pore size distributions of supports and membranes used in the experiment were all measured by the bubble-point method using a pore size analyzer (PSDA-30, Nanjing Gaoqian Functional Materials Technology Co., Ltd., Nanjing, China). The maximum, most probable and average pore sizes, together with the fraction of pores within the most probable pore-size interval, were all read directly from the measured pore size distribution. The corresponding cumulative distributions were used to obtain the pore sizes below which 10%, 50% and 90% of the pore population lie (d10, d50 and d90, respectively), the distribution width d90 − d10, the relative span (d90 − d10)/d50 and the skewness. Because d10, d50 and d90 are read from the cumulative distribution, they do not depend on the interval width used to plot the PSD, and the relative span is a dimensionless measure of the breadth of the distribution that can be compared directly between samples of different pore sizes. The same procedure was applied to the particle size distributions of the alumina powders measured by ImageJ, so that the particle size and the pore size distributions are described by identical parameters. All the experiments were repeated and measured three times, and the error bars represent the standard deviation of the three repeated measurements. The water fluxes of the supports and membranes were measured by a lab-made water flux testing platform, and the schematic diagram is shown in Figure 2. The feed water in the water container is pressurized with nitrogen. Four transmembrane pressures, 0.1, 0.2, 0.3 and 0.4 MPa, were applied in turn, and the water flux was measured at each of them. Each measurement was repeated three times. The value of the permeance obtained from these measurements is the flux normalized by the transmembrane pressure (L·m^−2^·h^−1^·bar^−1^).

### 2.4. Moisture Recovery Experiment

**Figure 3:**
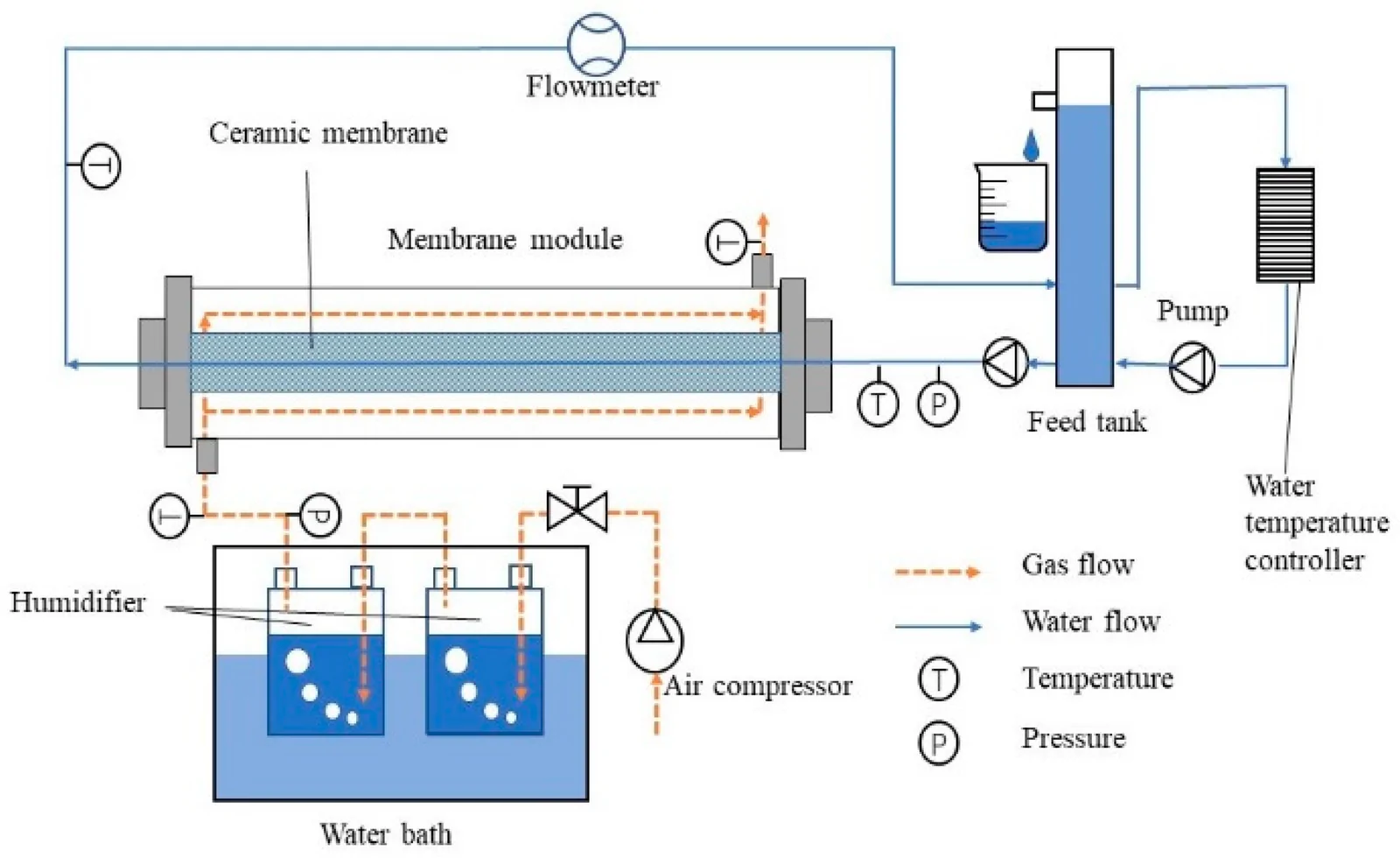
Schematic diagram of experimental platform.

The experimental platform uses a mixed gas flow of air and water vapor to simulate the flue gas. The schematic diagram is shown in Figure 3. The platform mainly consists of three parts, the simulated flue gas generation device, the membrane module, and the water temperature maintenance loop. The simulated flue gas generation device mixes water vapor with air and sends it to the shell side of the membrane module. The air flow rate is measured downstream of the humidifier, and both the temperature and the humidity of the gas reach equilibrium before the gas enters the membrane module. The simulated flue gas flows over the outer surface of the ceramic membrane within the membrane module. The moisture in the flue gas condenses and penetrates into the inner side of the ceramic membrane and is then recovered. The water flow and the flue flow within the membrane module flow in opposite directions. The water temperature maintenance loop is responsible for maintaining a constant water temperature at the inlet of the membrane module. The water level of the water tank is ensured to be the same with the highest overflow outlet before each experiment. When water is recovered, the volume of water flowing out from the overflow outlet is equal to the volume of the recovered water. And the volume of the overflowed water is measured to calculate the amount of recovered water.

To compare the moisture recovery performance of the ceramic membranes, moisture recovery flux and moisture recovery rate are used as evaluation indicators. The moisture recovery flux and moisture recovery rate are calculated according to Equations (1) and (2), respectively.

(1) $$J_{w}=\frac{\rho_{w}V_{w}}{10^{3}\times \Delta t\cdot S}$$

(2) $$\eta_{w}=\frac{J_{w}}{J_{v}}\times 100$$

where *J_w_* represents the moisture recovery flux (kg·m^−2^·h^−1^), *ρ_w_* is the density of pure water (kg·m^−3^), *V_w_* is the volume of overflow cooling water (L). Δ*t* is the duration of the experiment (h); *S* is the membrane surface area (m^2^), *J_v_* is the flow rate of water vapor entering the membrane module along with the gas flow (kg·m^−2^·h^−1^), which can be calculated by Equation (3):

(3) $$J_{v}=\frac{\rho_{a}d\cdot 60{\dot{V}}_{a}}{1000}$$

where *ρ_a_* represents the density of air (kg·m^−3^); *d* is the specific humidity of air at a certain temperature (g·kg^−1^); ${\dot{V}}_{a}$ is the air flow rate entering the humidifier (L·min^−1^).

## 3. Results and Discussion

### 3.1. Effect of Alumina Particle Content on Membrane Parameters

**Figure 4:**
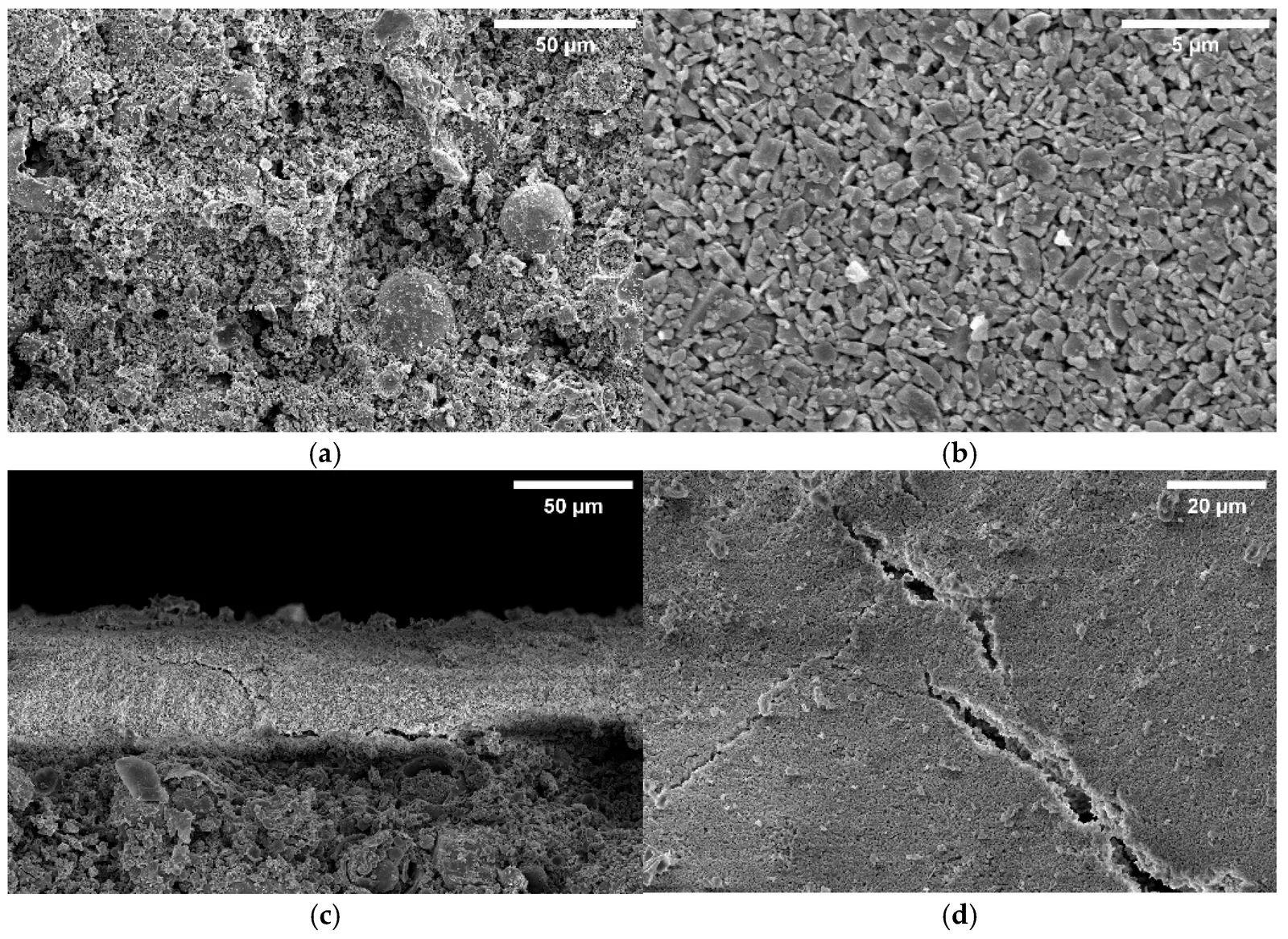
Surface and cross-section SEM images of CFA-based ceramic support and membranes. (**a**) Support surface; (**b**) membrane surface of 10 wt.% particle content; (**c**) membrane cross-section of 15 wt.% particle content; (**d**) membrane surface of 20 wt.% particle content.

**Figure 5:**
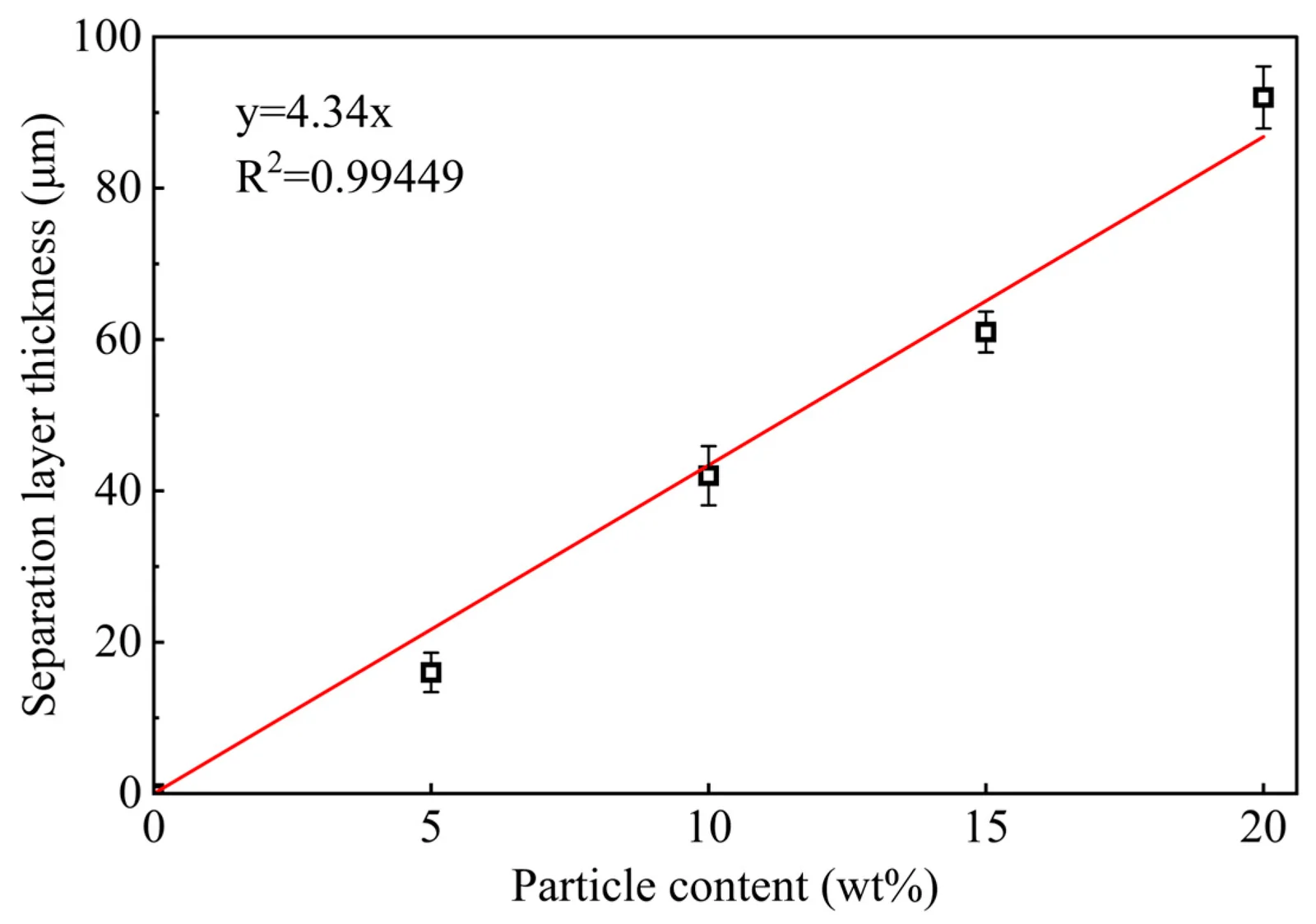
The relation between separation layer thickness and particle content. The solid line is the linear fit constrained to pass through the origin, y = 4.34x (R^2^ = 0.994, n = 4). Error bars represent the standard deviation of the thickness measured on the cross-sectional images.

The different contents of alumina particles play a crucial role in determining the surface morphology and structural parameters of the membranes during the separation-layer-formation process. To obtain the optimal content of alumina particles, alumina particles with an average size of 0.82 μm were selected, and five contents of alumina (5 wt.%, 10 wt.%, 15 wt.%, 20 wt.%, 25 wt.%) were prepared for the separation-layer-formation suspension. The effect of alumina content on the membrane parameters was investigated. Figure 4 shows the surface and cross-section SEM images of CFA-based ceramic support and membranes elaborated by separation-layer-forming suspension with different alumina particle content. As shown in Figure 4, when the particle content is not less than 15 wt.%, cracks will appear in different directions in the adhered separation layer. This is mainly because there is an upper limit for the thickness of the liquid film. When the liquid film is thicker than the upper limit, it will be subjected to a large lateral tensile stress and will be damaged during the drying process [48,49]. Therefore, the particle content should not exceed 10 wt.%. The thickness of the separation layer attached to the support is obtained by measuring the cross-sectional image of the membranes, and the results are shown in Figure 5. The figure indicates that, under the condition that other process parameters are fixed, the thickness of the separation layer will increase with the rise in particle content, and its thickness is nearly in a linear relationship with the particle content.

To describe this relationship quantitatively, a linear model constrained to pass through the origin, y = kx, in which y is the thickness of the separation layer (μm) and x is the alumina particle content (wt.%), was fitted to the thicknesses measured over the range 5–20 wt.%. An unconstrained fit gives an intercept that is not significantly different from zero (95% confidence interval −27.0 to +9.5 μm), which justifies the constrained model, and the constrained fit yields a slope of k = 4.34 ± 0.18 μm per wt.% (standard error, n = 4 points, 3 degrees of freedom; 95% confidence interval 3.76–4.92 μm per wt.%) with R^2^ = 0.994. The separation layer therefore thickens by approximately 4.3 μm for every additional weight percent of alumina in the coating suspension; the thickness at a particle content of 10 wt.% is 43 μm, which is used in the cost estimate in Section 3.4. The error bars in Figure 5 represent the standard deviation of the thickness measured on the cross-sectional images.

**Figure 6:**
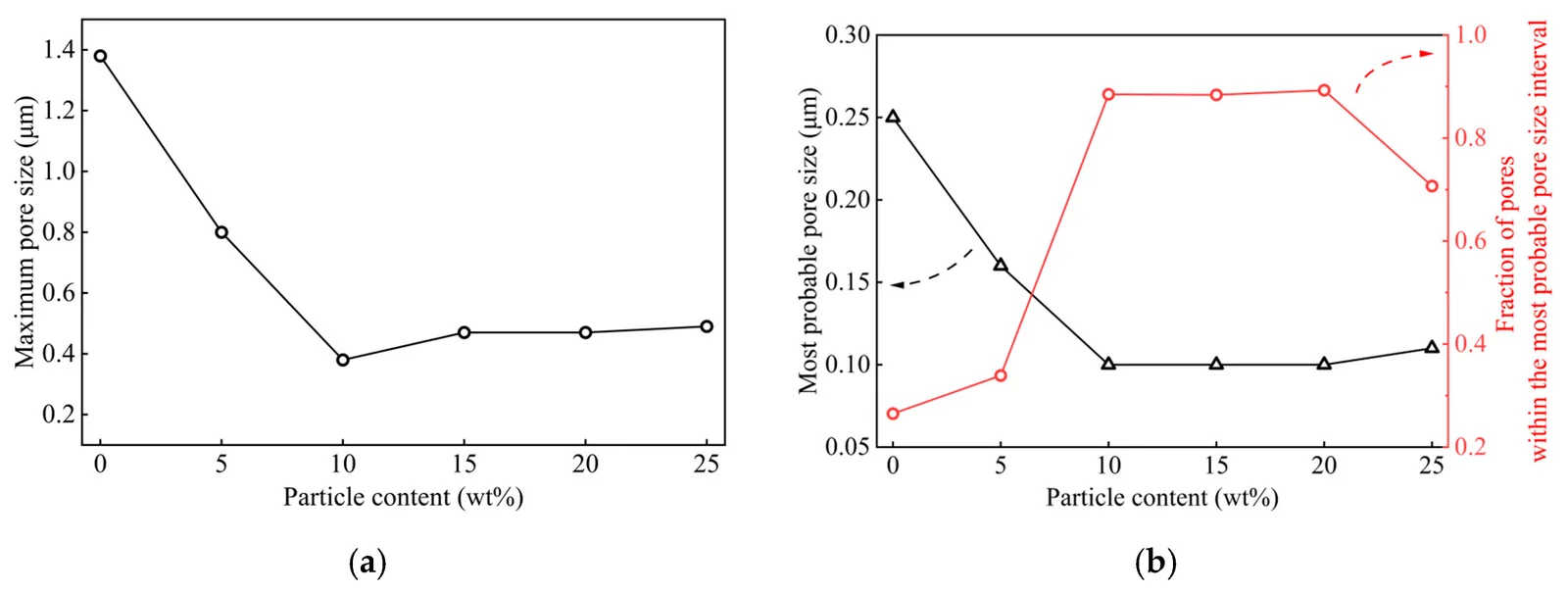
The relation between pore size and particle content. (**a**) Maximum pore size; (**b**) most probable pore size and the fraction of pores within the most probable pore-size interval.

Figure 6 shows the pore size distribution of the membranes under different particle contents. From Figure 6, it can be seen that when the alumina content is 5 wt.%, the maximum pore size of the membrane is significantly larger than that corresponding to the 10 wt.% alumina content, while the fraction of pores within the most probable pore-size interval is only 33.9%. The most probable pore size decreases from 0.25 μm for the support to 0.16 μm at a particle content of 5 wt.% and to 0.10 μm at 10 wt.%, while the fraction of pores within the most probable pore-size interval increases from 26.5% for the support to 33.9% at 5 wt.% and to about 88% at 10 wt.%; the maximum pore size falls from 0.80 to 0.38 μm over the same range (Figure 6a). The step between 5 and 10 wt.% is therefore a change in the character of the distribution rather than a gradual improvement, since most of the pore population is transferred into a single interval around 0.10 μm. A further increase in the content brings no improvement: between 10 and 20 wt.% the fraction stays at 88–89% while the maximum pore size rises again to about 0.47 μm, and at 25 wt.% the fraction decreases to about 70%.

These changes show that when the liquid film is coated on a support with a certain roughness, there is a critical thickness beyond which the liquid film can effectively cover the support and form a defect-free separation layer [50]. However, when the particle content is too low, the separation layer attached to the support is thin and cannot effectively cover the support, resulting in a larger maximum pore size and a broad pore size distribution. And the cracking of the layer will occur once the liquid film becomes thicker than the upper limit discussed above [48]. The two limits define a window rather than a single optimum content. The lower limit is set by the coverage of the support: at 5 wt.% the layer is too thin to close the largest pores, so that only 33.9% of the pore size lies within the most probable interval (Figure 6a,b). The upper limit is set by the cracking of the dried film: at 15 wt.% and above, cracks running in different directions appear in the adhered separation layer (Figure 4c,d). Between the two limits the fraction of pores within the most probable interval stays at 88–89% over 10–20 wt.%. 10 wt.% is thus the lowest content at which the distribution is already concentrated while the layer is still free of visible cracks. It should be noted that these limits are identified from the presence or absence of cracks in the SEM images, and that the density of defects has not been counted for each particle content; the choice of 10 wt.% therefore rests on this qualitative criterion rather than on a statistical one. Therefore, a separation-layer-forming suspension with a particle content of 10 wt.% should be used to apply the separation layer to ensure the integrity of the membranes and effectively reduce the maximum pore size.

### 3.2. Effect of Alumina Particle Size on Membrane Parameters

**Figure 7:**
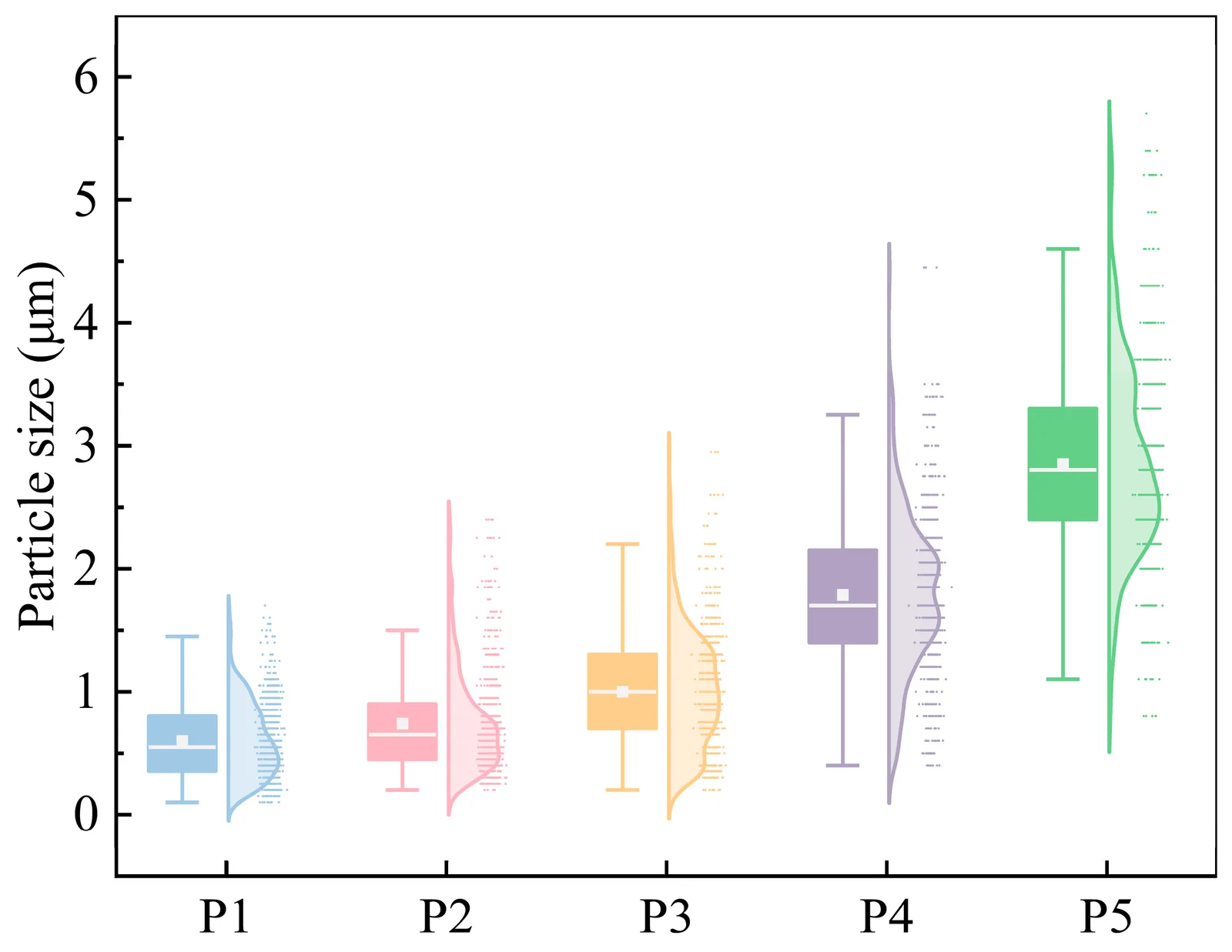
Alumina particle size distributions.

The particle size of alumina powder plays a decisive role in the structure and performance parameters of the membrane during the separation-layer-formation process. After determining the optimal content of alumina particles in the separation-layer-forming suspension, five different particle sizes of alumina particles were selected as separation-layer-forming particles to investigate their influence on the membrane parameters. The morphologies of the five different particle sizes of alumina particles were observed using a scanning electron microscope, and their particle size distributions were statistically analyzed using ImageJ. The obtained particle size distributions are shown in Figure 7, and the average particle size and the corresponding membrane numbers are shown in Table 2.

The parameters collected in Table 2 quantify the difference in the breadth of the five powders. The absolute width of the particle size distribution increases monotonically with the average particle size: d90 − d10 rises from 0.76 μm for P1 to 1.90 μm for P5, and the standard deviation from 0.30 to 0.99 μm. The relative span shows the opposite trend, decreasing from 1.44 for P1 to 0.72 for P5, that is, the coarser powders are broader in absolute terms but narrower relative to their own mean size. Since it is the average particle size that mainly determines the range of interparticle voids left behind when the particles pack, and hence the breadth of the pore size distribution of the separation layer, the average particle size is the quantity used in the discussion below.

**Figure 8:**
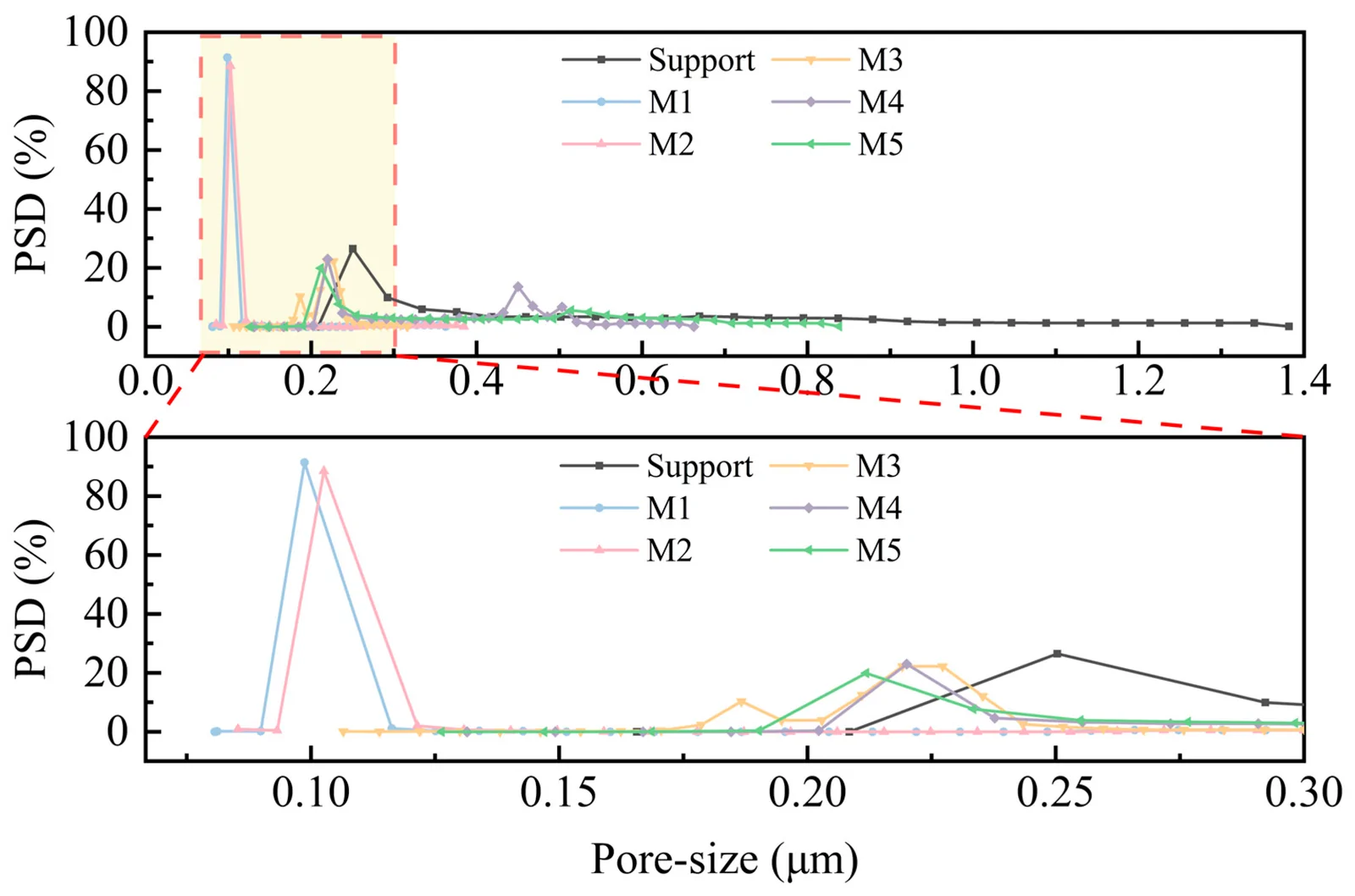
Pore size distribution of different membranes (red dotted line means enlarged display part).

Different particle sizes of alumina particles P1 to P5 were used to prepare the separation-layer-forming suspension with a particle content of 10 wt.%, and membranes were fabricated. The influence of particle size on the pore size distribution of the ceramic membranes was investigated. The results are shown in Figure 8 and Table 3. It can be seen that compared with the pore size distribution of the support, the pore size concentration degree of the five prepared membranes has been improved to varying levels. Particularly, compared with the support, the maximum pore size of the membranes has significantly decreased. However, the modifying effect of different alumina particle sizes on the pore size distribution varies greatly. When using larger alumina particles (P5, P4) to prepare the separation layer, the maximum pore size of the membranes obtained decreases with the reduction in particle size, but the most probable pore size and the average pore size of the membranes are similar to those of the support, and the fraction of pores within the most probable pore-size interval slightly decreases. When the alumina particle size used (P3, P2, P1) is small, the maximum pore size of the membrane stops decreasing, and the most probable pore size and the average pore size no longer show a significant decreasing trend after reducing to a certain value. When the fraction of pores within the most probable pore-size interval increases to about 90% after using the alumina particle size P2 and P1 which is relatively small, further reduction in the particle size produces no significant change.

During the process of the support being immersed in the separation-layer-forming suspension, the solvent of the separation-layer-forming suspension penetrates into the support pores under the action of capillary force, and the alumina particles in the separation-layer-forming suspension deposit on the surface of the support. At this time, under the action of the self-repair mechanism, more particles tend to deposit in the needle-like defect areas of the support [51]. In this study, the large pores of the support that deviate from the average pore size can be regarded as “needle-like defects”, so even if the particles used to construct the separation layer are relatively large, these particles will still preferentially accumulate in the large pores, reducing the maximum pore size. At the same time, from the particle size distribution of the particles, it can be seen that the larger the average particle size, the wider the particle size distribution is, and thus the pore size distribution of the constructed separation layer is also broader, and the fraction of pores within the most probable pore-size interval is also lower. With the reduction in the alumina particle size, the particle size distribution becomes narrower, the pore size distribution of the ceramic membrane becomes more concentrated, and the most probable pore size of the ceramic membrane also decreases.

The parameters in Table 3 show that the pore size distribution becomes more concentrated as the average particle size of the alumina powder decreases. For the two coarsest powders, P5 and P4 (average particle size 2.86 and 1.85 μm), the relative span decreases only to 1.16 and 0.76 and the median pore size remains close to that of the support, so that the separation layer acts mainly on the largest pores. A contraction of the whole distribution requires an average particle size of 1.11 μm (P3) or below: the relative span falls to 0.24 for M3 and to 0.16–0.17 for M2 and M1, the interval containing the central 80% of the pore population narrows from 0.80 μm for the support to 0.02 μm, and the fraction of pores within the most probable pore-size interval rises from 26.5% to 88.4–91.3%. The strong positive skewness of M1 and M2 (3.6) indicates that this concentration is achieved by suppressing the large-pore tail of the support rather than by removing it, so that the maximum pore size remains the parameter most sensitive to the pore structure of the support. How the support is modified can be read from the distributions themselves. With the two coarsest powders the main peak is almost unchanged with respect to the support while the large-pore end is displaced appreciably (maximum pore size and d90 falling from 1.38 and 1.02 μm to 0.84–0.66 and 0.66–0.50 μm), so that the layer acts selectively on the largest pores; with the finer powders the whole distribution is displaced towards smaller sizes (main peak at 0.22 and 0.10 μm, narrower span), so that the particles then govern the distribution once they are small enough to fill it. This reading is inferred from the shape of the distributions rather than observed directly.

Since the average pore sizes of the support and of the membranes lie between 0.11 and 0.41 μm, all of the samples studied here fall within the microfiltration range of the conventional classification of pressure-driven membrane processes, M1 and M2 lying at the boundary between microfiltration and ultrafiltration.

Although researchers have constructed the relationship between particle size and membrane pore size through experience [52], building mathematical models [53], and simulating particle stacking [43], the results presented in this study differ from the patterns presented by these studies. The reason for the difference lies in the fact that the above studies only focused on the corresponding relationship between particles and membrane pore size but did not consider the influence of the support. These relations were all obtained for the packing of the coating powder alone, and none of them consider the support. The results of this study show that they do not describe the membranes prepared here in two ways. First, the median pore size stops decreasing once the powder becomes coarse: it is 0.38 and 0.40 μm for M4 and M5, close to the median pore size of the support (0.41 μm), although the average particle size of P5 is nearly five times that of P1. Second, the pore size distribution of the membrane becomes wider as the particle size distribution of the powder becomes narrower: the relative span of the powder falls from 1.44 to 0.72, but that of the membrane rises from 0.16 to 1.16 and approaches that of the support (1.94). The packing of particles of unequal size, and the rearrangement that takes place while such a packing consolidates, has been simulated directly for powder packings with realistic interparticle interaction [54]. However, this study shows through experiments that the large pores of the support will be filled preferentially, causing the pore distribution to deviate from the prediction of the above models. CFA, as a cheap raw material for membrane production, has a large difference in particle size [33], and the maximum pore size of the support often deviates significantly from the average pore size. Therefore, the influence of the support on the pore size distribution of the ceramic membrane is more significant.

**Figure 9:**
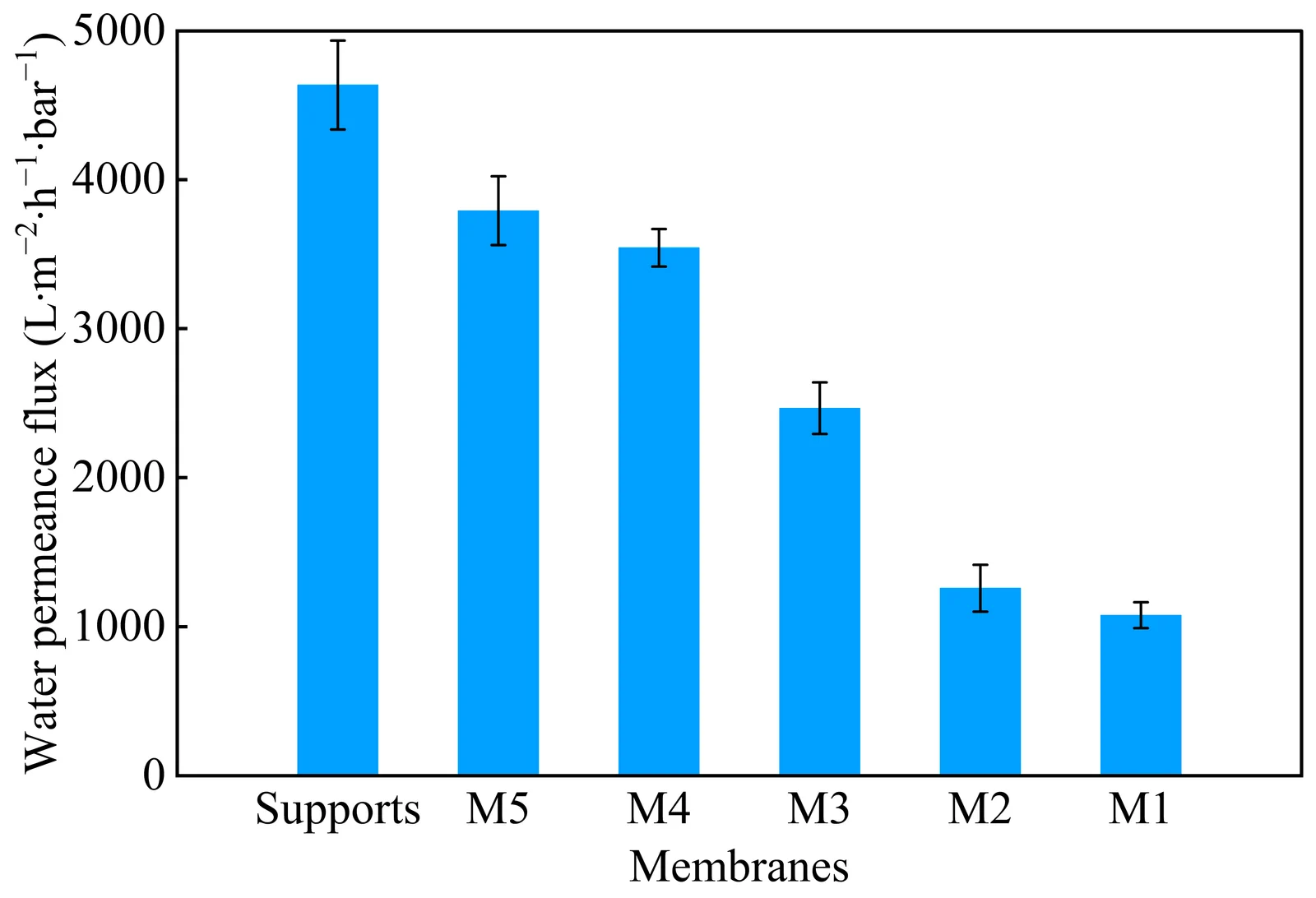
Water permeance fluxes of different membranes. Error bars represent the standard deviation of three repeated measurements.

The pure water flux of ceramic membranes made from different particle sizes of alumina is shown in Figure 9. As can be seen from Figure 9, the smaller the particle size of the alumina particles, the smaller the pore size of the separation layer prepared, and the lower the water flux of the ceramic membrane. The pure water fluxes of the membranes M1 and M2 with the narrowest pore size distributions are only 23.2% and 27.1% of that of the support, indicating that the resistance of the attached separation layer to water penetration is dominant.

### 3.3. Moisture Recovery Performance of Different Membranes

**Figure 10:**
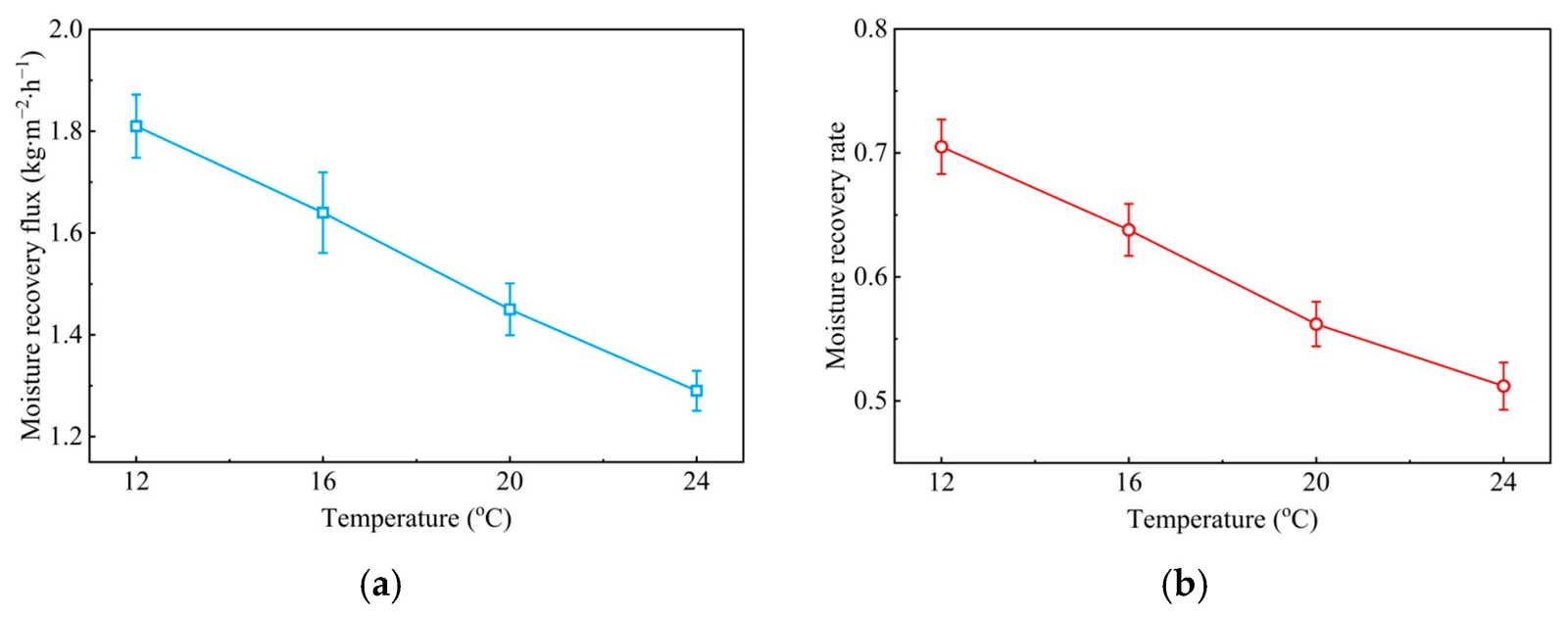
Moisture recovery performance of CFA-based membrane M2. (**a**) Moisture recovery flux with cooling water temperature; (**b**) moisture recovery rate with cooling water temperature.

To verify the feasibility of the CFA-based membrane with concentrated pore size distribution in the application of flue gas moisture recovery, an experimental study was conducted on its moisture recovery performance. Under the conditions of a flue gas temperature of 50 °C, a flue gas flow rate of 12 L·min^−1^, and a cooling water flow rate of 1.5 L·min^−1^, the moisture recovery performance of CFA-based membrane M2 was investigated. The cooling water temperature range of 12–24 °C was selected because it is commonly encountered in the cooling water systems of power plants and is generally adopted in the studies on membrane-based moisture recovery from flue gas [14,55]. The results are shown in Figure 10, which indicates that both the moisture recovery flux and the moisture recovery rate of the ceramic membrane decrease as the cooling water temperature rises. When the cooling water temperature increases from 12 °C to 24 °C, the moisture recovery flux and the moisture recovery rate decrease by 22.7–28.2% and 17–19 percentage points respectively. A higher cooling water temperature will increase the outer wall temperature of the membrane, resulting in a decrease in the condensation rate of water vapor in the flue gas within the membrane module, thereby reducing the moisture recovery flux. The moisture recovery flux can reach 1.81 kg·m^−2^·h^−1^ while the moisture recovery rate can reach 70.5%. The membrane prepared benefits from the use of a support with a large water permeance flux. While achieving a reduced pore size and increased degree of pore size concentration, it ensures good moisture recovery performance and is expected to reduce the manufacturing cost of TMC.

The moisture recovery test was carried out with membrane M2 only, since the purpose was to verify the feasibility of the CFA-based membrane rather than to screen the five membranes. M2 was selected because it has the smaller pore size and the narrower pore size distribution compared to M3–M5, and relatively higher water permeance compared to M1. The same pore size and the same degree of pore size concentration are thus obtained at a lower penalty in water permeance. This reflects the trade-off between pore size concentration, water permeance and moisture recovery that is inherent to the separation layer. A smaller and more concentrated pore size improves the quality of the recovered water, since it blocks the transmembrane permeation of soluble pollutants in the flue gas [23], but it also increases the permeation resistance of the membrane (Figure 9) and, if the pores become too small, thickens the condensate film on the flue gas side and thus raises the mass- and heat-transfer resistances [21]. Pore size concentration and a small pore size are therefore obtained at the cost of water permeance, and among the membranes prepared in this work this trade-off is the most favorable for M2.

Table 4 compares the moisture recovery performance of M2 with that reported for ceramic membranes in this application; the operating conditions are listed for each entry, since both indicators depend on them. At a flue gas temperature of 50 °C, the moisture recovery flux of M2 is 1.81 kg·m^−2^·h^−1^ at a cooling water temperature of 12 °C, which is higher than the 1.50–1.66 kg·m^−2^·h^−1^ reported for a fly ash-based ceramic membrane tested in a module of the same geometry [55], although the mean pore size of M2 (0.11 μm) is more than an order of magnitude smaller than that of the latter membrane (5–9 μm). Narrowing pore size distribution thus reduces the water permeance, but it does not make the membrane inferior to the reported ceramic membrane in the amount of water recovered.

### 3.4. Economical Analysis

The use of coal fly ash as the main raw material of the support is the basis of the low-cost claim of this work, whereas the separation layer is formed from α-Al_2_O_3_, which is a much more expensive material. Whether the separation layer undermines the low-cost positioning therefore needs to be quantified. In this section, the raw-material cost of the CFA-based membrane is estimated and compared with that of alumina-based ceramic membranes.

The estimate accounts for raw materials only and is expressed per square meter of membrane area; the energy, labor, equipment, module assembly and profit involved in fabrication are not included, so the values obtained represent a lower bound of the production cost. The unit prices of coal fly ash (8.5 USD·t^−1^) and α-Al_2_O_3_ (820 USD·t^−1^) are taken from the cost analysis of fly-ash-based ceramic membrane supports previously reported by our group [55]. The consumption of each material per unit area is derived from the geometry given in Table 1, the open porosity of the CFA-based support (42.0%) measured in our previous work [47], and the separation-layer thickness measured in the present work (43 μm at a particle content of 10 wt.%, Figure 4). The organic additives are assumed to be used at a combined loading of 10 wt.% relative to coal fly ash (3 wt.% dextrin, 2 wt.% CMC and 5 wt.% glycerin); these additives are burnt out during sintering and do not remain in the final support, but they are consumed as raw materials and are therefore included.

As shown in Table 5, the total raw-material cost of the membrane is only about 0.4 USD·m^−2^. The separation layer is the only part of the membrane that uses alumina, and it accounts for about 25% of this cost, of which the α-Al_2_O_3_ powder itself contributes about 20%. This limited contribution is a direct consequence of the fact that the separation layer is a thin coating: at a thickness of 43 μm and an assumed porosity of 40%, only about 0.10 kg·m^−2^ of α-Al_2_O_3_ is required, whereas an all-alumina support of the same geometry would consume about 4.0 kg·m^−2^. The use of an α-Al_2_O_3_ separation layer is therefore compatible with the low-cost positioning of the membrane.

The cost reduction originates mainly from the replacement of the support material. In commercial alumina ceramic membranes, the manufacturing components contain about 90 wt.% α-Al_2_O_3_ [55], the price of which is about two orders of magnitude higher than that of coal fly ash. On the basis of the unit prices given above, the ceramic body of an all-alumina support with the same geometry would cost about 3.3 USD·m^−2^, roughly 165 times that of the CFA-based support (0.02 USD·m^−2^). Even when the organic additives and the separation layer are included, the raw-material cost of the all-alumina membrane is estimated to be about 3.7 USD·m^−2^, approximately nine times that of the membrane prepared in this work. In addition, the support and the membrane are sintered at 1150 °C and 1100 °C, respectively, which is lower than the temperature of up to 1500 °C usually required for alumina ceramic membranes [55], further reducing the energy consumption of fabrication. A further reduction is possible if the separation layer is co-sintered with the support, since a single thermal treatment removes an entire firing step [57]. The same principle of replacing a high-purity oxide with a cheap mineral precursor has been applied to other ceramic systems: mullite-titania hollow fibers have been prepared as a low-cost alternative [58], SiC membranes have been sintered at a reduced temperature by using a residue as sintering aid [59], and beta-Sialon hollow fibers with a low thermal conductivity have been developed for thermally driven processes [60].

Table 6 compares the estimated cost of the membrane with the fabrication costs reported for other ceramic membranes. Although the present estimate covers raw materials only and cannot be compared directly with the reported fabrication costs, which include capital and operating expenditure, the value obtained is consistent with the lower bound of the cost range reported for membranes derived from waste and natural materials (3.4–250 USD·m^−2^) and is far below the range reported for membranes fabricated from commercial-grade starting materials such as α-Al_2_O_3_, ZrO_2_ and SiC (100–3000 USD·m^−2^) [61].

In the last row of Table 6, the specific material cost is defined as the estimated raw-material cost per unit membrane area divided by the moisture recovery flux and thus represents the material cost required to recover one kilogram of water per hour; for the alumina-based membrane an equivalent moisture recovery flux is assumed, so that only the difference in material cost is accounted for. On this basis, water is recovered by the CFA-based membrane at a material cost of about 0.22 USD·h·kg^−1^, roughly one ninth of that of the alumina-based membrane. Thus, the use of CFA as the raw material for the ceramic membrane in flue gas moisture recovery significantly reduces the production cost of the TMC.

## 4. Conclusions

A coal-fly-ash-based (CFA-based) support was prepared, and on this basis, low-cost ceramic membranes with a concentrated pore size distribution were successfully fabricated using alumina particles of different sizes. Meanwhile, the performance of CFA-based membranes in the flue gas moisture recovery was investigated. The following conclusions were drawn:

(1) The preparation of ceramic membranes requires that the separation-layer-forming suspension have an appropriate content of alumina. A large content will cause cracks in the ceramic membranes, while a small content cannot effectively concentrate the broad pore size distribution of the support.

(2) The pore size and pure water flux of the ceramic membranes decrease as the size of the alumina particles used for forming the separation layer decreases. When using larger alumina particles, during the process of the separation-layer-forming suspension wetting the support, the larger pore sizes on the support will be filled preferentially, causing the maximum pore size of the membrane to decrease earlier than the most probable pore size. This indicates that the pore sizes of the ceramic membranes are not only related to the particle size of the alumina particles but are also affected by the support.

(3) Under the operating conditions investigated here, the moisture recovery flux can reach 1.81 kg·m^−2^·h^−1^ while the moisture recovery rate comes up to 70.5%. Moisture is recovered by the CFA-based membrane at a material cost of about 0.22 USD·h·kg^−1^, roughly one-ninth of that of the alumina-based membrane (2.0 USD·h·kg^−1^). The results indicate that the membrane has good potential in maintaining high moisture recovery performance and reducing the membrane production cost based on low-cost ceramic membranes, which is expected to be conducive to the industrial application of transport membrane condensers.

The present study was aimed at the preparation of a low-cost ceramic membrane with a concentrated pore size distribution, and the membrane was assessed in terms of the amount of water recovered rather than the quality of that water. For ceramic membranes of different pore sizes, a smaller pore size has been reported to reduce the water recovery while improving the quality of the recovered water, because the transmembrane permeation of soluble pollutants is suppressed [18]. The rejection of pollutants was not investigated in this work. Membranes covering a range of pore size distributions can therefore be compared in follow-up work in terms of both the quantity and the quality of the water recovered, so that this trade-off can be quantified and the most favorable pore size distribution identified. The long-term stability of the membrane and its resistance to fouling, which are critical for industrial flue gas applications with acidic and particulate-laden streams, were not evaluated in this work and will be the subject of follow-up work with flue gas containing acidic gases and particulate matter.

## Figures and Tables

**Table 1:** Geometrical parameters of CFA-based support and membranes.

Parameters	Value
Inner size	8 mm
External size	12 mm
Length	79 cm
Effective membrane area	0.0294 m^2^

**Table 2:** Alumina particle size and corresponding composite membrane numbers.

Numbers of Alumina Particle	Average Particle Size (μm)	d10 (μm)	d50 (μm)	d90 (μm)	d90 − d10 (μm)	Relative Span	Numbers of Obtained Membrane
P1	0.58	0.22	0.53	0.98	0.76	1.44	M1
P2	0.82	0.32	0.63	1.25	0.93	1.48	M2
P3	1.11	0.36	0.92	1.53	1.17	1.26	M3
P4	1.85	0.92	1.69	2.50	1.58	0.94	M4
P5	2.86	1.90	2.64	3.80	1.90	0.72	M5

**Table 3:** Variation in membrane pore size with particle size.

Membranes	Maximum Pore Size (μm)	Most Probable Pore Size (μm)	Fraction of Pores Within the Most Probable Pore-Size Interval	Average Pore Size (μm)	d10 (μm)	d50 (μm)	d90 (μm)	Relative Span	Skewness
Ceramic support	1.38	0.25	26.5%	0.41	0.22	0.41	1.02	1.94	0.92
M5	0.84	0.21	19.9%	0.40	0.20	0.40	0.66	1.16	0.37
M4	0.66	0.22	23.0%	0.39	0.21	0.38	0.50	0.76	0.14
M3	0.32	0.22	69.0%	0.23	0.18	0.22	0.23	0.24	0.42
M2	0.38	0.10	88.4%	0.11	0.09	0.10	0.11	0.17	3.66
M1	0.36	0.10	91.3%	0.11	0.09	0.10	0.11	0.16	3.60

**Table 4:** Comparison of the moisture recovery performance of the CFA-based membrane M2 with that of ceramic membranes reported by other studies.

Membrane (Average Pore Size)	Flue Gas Temperature (°C)	Flue Gas Flow Rate (L·min^−1^)	Cooling Water Temperature (°C)	Cooling Water Flow Rate (L·min^−1^)	Moisture Recovery Flux (kg·m^−2^·h^−1^)	Moisture Recovery Rate (%)	Ref.
CFA-based membrane M2 (0.11 μm)	50	12	12–24	1.5	1.81 (at 12 °C)	70.5 (at 12 °C)	This work
Talcum–fly-ash-based membrane (5–9 μm)	50	9	15–25	1.0	1.66 (15 °C)–1.50 (25 °C)	79.7 (15 °C)–73.0 (25 °C)	[55]
Commercial α-Al_2_O_3_ membrane tubes (30/50/200 nm)	45–60	5–20	15–30	0.5–2	1.7–4.82	up to 80.3	[23]
Commercial α-Al_2_O_3_ membrane (0.22 μm)	38.5–60	14.5	22	0.33	1.16–6.09	46–72	[56]

**Table 5:** Estimated raw-material cost of the CFA-based membrane (per square meter of membrane area).

Item	Unit Price (USD·kg^−1^)	Consumption (kg·m^−2^)	Cost (USD·m^−2^)
Coal fly ash (support body)	0.0085	2.50	0.02
Organic additives (dextrin, CMC, glycerin)	1.1	0.25	0.28
α-Al_2_O_3_ (separation layer)	0.82	0.10	0.08
SHMP and PVA (suspension additives)	1.6	0.01	0.02
Total (support and separation layer)	—	—	0.4

— indicates not applicale.

**Table 6:** Comparison of the membrane cost and the cost-normalized moisture recovery performance.

Indicator	This Work (CFA-Based, M2)	Alumina-Based Ceramic Membrane
Main material of the support	Coal fly ash	α-Al_2_O_3_ (90 wt.% of the body) [55]
Sintering temperature (°C)	1100–1150	up to 1500 [31]
Estimated raw-material cost (USD·m^−2^)	0.4	3.7
Reported fabrication cost (USD·m^−2^)	3.4–250 (waste-derived) [32]	100–3000 (commercial materials) [61]
Mean pore size (μm)	0.11 (M2, MF range)	0.1–1 (MF range)
Moisture recovery flux (kg·m^−2^·h^−1^)	1.81	1.81
Specific material cost (USD·h·kg^−1^)	0.22	2.0

## Data Availability

The data presented in this study are available on request from the corresponding author.
